# Development and validation of a nomogram for predicting frailty among older adults with multimorbidity in the emergency department

**DOI:** 10.3389/fmed.2026.1781116

**Published:** 2026-04-14

**Authors:** Jingting Zhang, Menghui Ma, Xiaoguang Xie, Dou Chu, Jinzhong Hao, Weiqin Zhang, Shuyan Deng, Peng Ouyang, Zhongqi Guo

**Affiliations:** 1 Emergency Department, Shanghai University of Traditional Chinese Medicine, Shenzhen Hospital, Shenzhen, Guangdong, China; 2 Nursing Department, Shanghai University of Traditional Chinese Medicine, Shenzhen Hospital, Shenzhen, Guangdong, China; 3 ICU Department, Shanghai University of Traditional Chinese Medicine, Shenzhen Hospital, Shenzhen, Guangdong, China

**Keywords:** emergency department, frailty, multimorbidity, nomogram, older adults

## Abstract

**Objective:**

Frailty is common among older adults with multimorbidity presenting to emergency departments (EDs), yet rapid, practical tools for identifying current frailty status in this setting remain limited. We aimed to identify factors associated with frailty and to develop a clinically applicable nomogram for frailty screening in the ED.

**Methods:**

We conducted a hospital-based cross-sectional study including 486 multimorbid adults aged ≥60 years in a tertiary ED. Frailty was assessed using the 5-item FRAIL scale. Candidate variables were pre-specified and screened using the least absolute shrinkage and selection operator (LASSO) regression, and the variables selected by LASSO were then entered into a multivariable logistic regression to develop a model to identify frailty status. The model was developed using the full dataset (*n* = 486) and internally validated using 1,000 bootstrap resamples. Discrimination was evaluated using receiver operating characteristic (ROC) curves and the area under the curve (AUC). Calibration was assessed using calibration plots, the calibration intercept, the calibration slope, and the Brier score. Clinical utility and potential impact were assessed using decision curve analysis (DCA) and clinical impact curves (CIC).

**Results:**

The prevalence of frailty was 32.3%. Six independent factors were retained in the nomogram: number of medications, fall risk, Charlson Comorbidity Index (CCI), nutritional status, anxiety, and ADL dependence. The nomogram demonstrated good discrimination (AUC = 0.877) and remained after 1,000 bootstrap resamples (AUC = 0.883). Calibration was acceptable, with intercepts of −0.028 and slopes of 0.926; Brier scores were 0.133 and 0.140 for the uncalibrated and calibrated logistic models, respectively. The Hosmer–Lemeshow test indicated good model fit (*χ*^2^ = 8.876, *p* = 0.262). DCA and CIC indicated favorable clinical utility and good accuracy in identifying frailty.

**Conclusion:**

The nomogram may be an effective tool for assessing current frailty status among multimorbid older adults in an emergency setting. It could support routine frailty screening, facilitate communication among healthcare professionals, and inform individualized care planning.

## 1 Introduction

With the accelerating trend of population aging, emergency departments (EDs) are increasingly challenged by the rising number of older adults living with multimorbidity and frailty (1). Multimorbidity is defined as the coexistence of two or more long-term diseases (2), substantially increasing clinical complexity and predisposing patients to frailty (3). Frailty, a multidimensional geriatric syndrome characterized by reduced stress tolerance and heightened vulnerability to adverse outcomes, has been consistently linked to falls, disability, hospitalization, and prolonged ED stays (4–6). Moreover, older adults with both frailty and multimorbidity face significantly higher mortality risk compared with those who are frail alone (7). Therefore, timely identification of current frailty status in older patients presenting to the emergency department is important for supporting prompt assessment and guiding individualized clinical management.

In recent years, research on frailty prediction models has expanded steadily. A systematic review by Kong et al. (8) synthesized 22 models developed in community and hospital settings and identified age, cognitive function, self-rated health, functional limitations, and depressive symptoms as common factors, reflecting the multidimensional determinants of frailty. However, issues such as insufficient handling of missing data and limited calibration reporting constrain their clinical applicability. Evidence explicitly focused on older adults with multimorbidity who bear a disproportionately high frailty burden remains scarce. Huang et al. (9) developed a model for hospitalized multimorbid older adults, identifying age, BMI, education level, physical activity, medication burden, and comorbid CHF, COPD, and CCVD as key factors with frailty, with moderate discrimination (AUC = 0.736). These findings collectively highlight the need for more rigorous, clinically practical models to support frailty screening and identification in multimorbid populations, particularly in emergency care settings, where timely assessment is critical.

A nomogram is a graphical representation of a prediction model that integrates multiple predictive variables to estimate specific clinical assessments accurately (10). However, most existing frailty prediction models have been developed using community-dwelling or general inpatient populations (9, 11–14). They therefore fail to meet the assessment needs of multimorbid older adults in emergency settings, where rapid changes in clinical status and limited decision-making time are common (1). Consequently, developing a frailty prediction tool suitable for the emergency department (ED) and that fully accounts for the characteristics of multimorbidity has substantial clinical significance. To address these clinical needs and research gaps, this study aims to develop and validate a nomogram using routinely obtained ED assessment indicators to identify current frailty status in older adults with multimorbidity. As a simple and efficient adjunct screening tool, the model may support timely frailty assessment and individualized clinical management in the ED.

## 2 Materials and methods

### 2.1 Study design and participants

This hospital-based cross-sectional study was conducted in one tertiary hospital in Shenzhen, China. Data were collected for 6 months from September 2024 to February 2025. The inclusion criteria were as follows: (1) Older patients (60 years and above) who visited the ED in the selected hospital. (2) Eligible participants were those with multimorbidity or at least two chronic diseases. (3) Patients were assessed for their frailty status via a 5-item FRAIL scale during their ED visit. (4) The participants were Chinese. The exclusion criteria were as follows: (1) Patients with severe cognitive impairments (e.g., dementia) who hindered their assessment participation. (2) Individuals with critical illnesses or at the terminal stage. (3) Patients who declined participation or could not provide informed consent. In this study, multimorbidity was defined as the presence of any two or more, according to the 10th revision of the International Classification of Diseases (ICD-10) (15), this study investigated 12 types of chronic diseases, such as hypertension (HTN), diabetes mellitus (DM), coronary heart disease (CHD), chronic heart failure (CHF), chronic obstructive pulmonary disease (COPD), chronic cerebrovascular disease (CCVD), bone and joint disease, chronic kidney disease (CKD), chronic gastrointestinal disease, chronic liver disease, cerebral infarction (CI), and cancer.

### 2.2 Sampling method

Sample size was estimated using an events-per-variable (EPV) approach for logistic regression. We anticipated including 6–10 candidate predictors in the final model. Following the conventional 10-EPV rule (16), and the estimated prevalence of frailty among older adults in emergency at approximately 40% (17), the minimum sample size was calculated as: *N* = 10 × P ÷ 40% × (1 + 20%). Where p denotes the number of predictors, and 20% accounts for potential attrition/missing data. This yielded an estimated range of approximately 180 participants for 6 predictors and 300 participants for 10 predictors. Ultimately, 486 participants were enrolled, exceeding the minimum requirement. Penalized regression approaches such as LASSO have been shown to achieve acceptable predictive performance under relatively lower EPV conditions by reducing overfitting and improving model calibration (18). These findings supported the use of LASSO for predictor selection in the present study. Model development and reporting followed the TRIPOD guidelines.

### 2.3 Measurements

#### 2.3.1 Sociodemographic characteristics

Data were collected using a structured questionnaire administered through standardized interviews, supplemented with information extracted from the hospital information system (HIS). Patient characteristics comprised 11 variables: age, sex, body mass index (BMI) category, education level, living status, income, hobbies, smoking status, alcohol consumption, occupational status, and number of medications. Laboratory parameters included six routinely measured indicators: white blood cell count (WBC), red blood cell count (RBC), hemoglobin (HGB), platelet count (PLT), sodium (Na), and creatinine (Cr).

#### 2.3.2 Frailty

Frailty status was assessed using the 5-item FRAIL scale, which includes fatigue, resistance, ambulation, illness, and weight loss within the past year (19). Each item is scored 0/1, yielding a total score of 0–5. Participants scoring 3–5 were classified as frail, whereas those scoring 0–2 were classified as non-frail (including pre-frail individuals), a classification commonly used when a binary frailty outcome is required for predictive modeling. The Chinese version of the FRAIL scale has demonstrated cross-cultural equivalence and acceptable measurement properties, including satisfactory diagnostic accuracy against the Fried frailty phenotype (AUC = 0.91) and good test–retest reliability (ICC = 0.708) (20).

#### 2.3.3 Charlson comorbidity index (CCI)

The Charlson Comorbidity Index (CCI) is a weighted comorbidity measure developed initially to quantify disease burden and predict mortality (21). It comprises 19 comorbid conditions, each assigned a weight of 1, 2, 3, or 6, and the total score is obtained by summing these weights. For the age-adjusted CCI, additional points are assigned for age starting at 50 years, with 1 point added for each decade above 50 (22). The CCI has also been validated for predicting mortality in Chinese older adults (23).

#### 2.3.4 Fall

The Morse Fall Scale (MFS) is a widely used fall risk assessment tool for hospitalized and elderly patients (24). Six key factors contributing to fall risk are evaluated: history of falls, secondary diagnoses, use of assistive devices, IV therapy, gait stability, and mental status. Each factor is assigned a score ranging from 0 to 125, where higher scores indicate greater fall risk. A score of 0–24 is low risk. 25–44 is moderate risk. A score of ≥45 is high risk. The Cronbach’s *α* coefficient of the MFS is 0.96, the sensitivity is 0.78, and the specificity is 0.83 (25).

#### 2.3.5 Activities of daily living (ADL) dependent

Activities of daily living (ADL) were assessed using the Barthel Index (26), which evaluates functional performance across domains including feeding, bathing, personal grooming, dressing, bowel and bladder continence, toileting, transferring (e.g., from bed to chair), walking, turning in bed, and stair climbing. The total score ranges from 0 to 100, with scores ≤40 indicating severe dependence, 41–60 indicating moderate dependence, 61–99 indicating mild dependence, and 100 indicating no reliance. We used the Chinese version, and Cronbach’s alpha was 0.95 (27).

#### 2.3.6 Nutritional status

The Mini Nutritional Assessment (MNA) is a widely used tool for evaluating the nutritional status of elderly individuals, particularly in clinical and geriatric settings (28). It helps identify malnutrition or the risk of malnutrition to enable early intervention. The MNA consists of questions and measurements assessing nutritional intake, weight changes, mobility, psychological stress, BMI, and other nutrition-related factors. A score of ≥24 points indicate a normal dietary status. A score of 17–23.5 points indicate a risk of malnutrition. Those with <17 points are malnourished. It has been widely used in many countries, and its reliability and validity have been evaluated in various clinical settings (29).

#### 2.3.7 Anxiety

The GAD-7 is a widely used questionnaire designed to assess generalized anxiety disorder (GAD) and the severity of anxiety symptoms (30). It consists of 7 questions, each scored on a 0–3 scale, resulting in a total score range of 0–21. 0–4 is minimal anxiety. 5–9 indicates mild anxiety. Scores of 10–14 indicate moderate anxiety. 15–21 indicates severe anxiety. A meta-analytic study indicated that the GAD-7 score demonstrated good diagnostic performance, with a sensitivity of 0.83 and a specificity of 0.84 (31).

#### 2.3.8 Depression

Depressive symptoms were assessed using the Chinese version of the 15-item Geriatric Depression Scale (GDS-15). Each item is scored dichotomously (yes/no), yielding a total score of 0–15, with higher scores indicating more severe depressive symptoms. A cutoff score of ≥8 was used to define the presence of depressive symptoms. The Chinese version of GDS-15 has demonstrated good reliability (Cronbach’s *α* = 0.80) and test–retest reliability (0.73) (32).

### 2.4 Study procedure

After written informed consent was obtained, trained staff conducted face–to–face interviews in the ED. Participants were consecutively recruited during their ED visits, with a maximum of five individuals approached daily to ensure data quality and adequate time for assessment. Recruitment information was provided verbally and in writing, detailing the purpose of the study, data confidentiality, and voluntary participation. All the questionnaires were administered in the Chinese language. When completing the tools, participants were interviewed in a quiet room or bedside, depending on their condition, and assistance was provided as needed. Each interview was conducted individually by one trained nurse at a time to ensure consistency and data quality. A total of six nurses participated in the data collection process on a rotational basis and supported participants with difficulties due to vision, literacy, or cognitive challenges. In addition, retrospective data were extracted from medical records to complement the questionnaire responses. Finally, two people entered the collected data into Excel to ensure consistency.

### 2.5 Statistical analysis

The data were analyzed using SPSS 29.0 (IBM Corp., Armonk, NY, United States) and R4.3.3. Continuous variables were assessed for normality and are presented as mean ± standard deviation (SD) or median (interquartile range, IQR), as appropriate. Group comparisons were performed using the independent-samples *t*-test for normally distributed continuous variables and the Mann–Whitney *U* test for non-normally distributed variables. Categorical variables are presented as counts and percentages and were compared using the *χ*^2^ test or Fisher’s exact test, as appropriate. Multivariable logistic regression was used to identify factors independently associated with frailty among older adults with multimorbidity. All tests were two-sided, and *p* < 0.05 was considered statistically significant.

Missingness was low across variables (0–3.3%), including BMI (0.6%), number of medications (0.4%), WBC/RBC/HGB/PLT (each 1.0%), sodium (1.2%), and creatinine (3.3%). Missing values in predictors were handled using multiple imputation by chained equations (20 imputations), with all candidate predictors and frailty status included in the imputation models; results were pooled using Rubin’s rules. Prespecified candidate predictors were entered into LASSO regression with 10-fold cross-validation to select variables. Multicollinearity was assessed using variance inflation factors (VIF), and selected variables were then entered into a multivariable logistic regression model. A nomogram was developed based on the final model. Internal validation was conducted using bootstrap resampling (1,000 repetitions). Model discrimination and calibration were assessed using the AUC, calibration plots, and the Brier score. Clinical utility was evaluated using decision curve analysis (DCA) and clinical impact curves (CIC). DeLong’s test was used to compare ROC curves.

### 2.6 Ethical approval

This study was approved by the ethical committee of Shenzhen Luo Hu Traditional Chinese Hospital, in accordance with the Helsinki Declaration for medical research involving human participants (No: 2024-LHQZYYYXLL-KY-063). All participants provided informed consent before data collection. Participation was voluntary, and all the data were handled confidentially. Personal data were de-identified, coded, and retained for 2 years after the study was completed before destruction.

## 3 Results

### 3.1 Participant characteristics

In this study, a total of 486 participants were included, comprising 258 females (53.09%) and 228 males (46.91%), with a mean age of 71.00 years. The prevalence of frailty was 32.3%. Detailed baseline characteristics were presented in Table 1.

**Table 1:** Baseline characteristics of the participants.

Variables	Total (*n* = 486)	Non-frailty (*n* = 329)	Frailty (*n* = 157)	*P*
Age [year, M (P25, P75)]	71.00 [65.00; 77.00]	69.00 [64.00; 75.00]	75.00 [68.00; 83.00]	<0.001
Gender				0.299
Female	258 (53.09%)	180 (54.71%)	78 (49.68%)	
Male	228 (46.91%)	149 (45.29%)	79 (50.32%)	
BMI [M (P25, P75)]	22.67 [20.80; 24.12]	22.60 [21.10; 24.34]	22.68 [20.00; 23.93]	0.064
Smoking status				0.802
No	434 (89.30%)	293 (89.06%)	141 (89.81%)	
Yes	52 (10.70%)	36 (10.94%)	16 (10.19%)	
Alcohol consumption				0.100
No	240 (49.38%)	154 (46.81%)	86 (54.78%)	
Yes	246 (50.62%)	175 (53.19%)	71 (45.22%)	
Education				0.001
Primary	77 (15.84%)	38 (11.55%)	39 (24.84%)	
Middle	214 (44.03%)	152 (46.20%)	62 (39.49%)	
Secondary	144 (29.63%)	102 (31.00%)	42 (26.75%)	
Graduate	42 (8.64%)	28 (8.51%)	14 (8.92%)	
Postgraduate	9 (1.85%)	9 (2.74%)	0 (0%)	
Living status				0.091
Live alone	20 (4.12%)	17 (5.17%)	3 (1.91%)	
Live with family	466 (95.88%)	312 (94.83%)	154 (98.09%)	
Hobbies				<0.001
No	236 (48.56%)	180 (54.71%)	56 (35.67%)	
Yes	250 (51.44%)	149 (45.29%)	101 (64.33%)	
Income				0.006
Poor	130 (26.75%)	86 (26.14%)	44 (28.03%)	
Adequate	192 (39.51%)	145 (44.07%)	47 (29.94%)	
Rich	164 (33.74%)	98 (29.79%)	66 (42.04%)	
Occupational status				0.003
Working	89 (18.31%)	72 (21.88%)	17 (10.83%)	
Not working	397 (81.69%)	257 (78.12%)	140 (89.17%)	
Number of medications [M (P25, P75)]	3.00 [2.00; 4.00]	3.00 [2.00; 4.00]	4.00 [3.00; 5.00]	<0.001
CCI [M (P25, P75)]	4.00 [3.00; 5.00]	3.00 [3.00; 4.00]	4.00 [4.00; 5.00]	<0.001
Fall risk				<0.001
Low fall risk	99 (20.37%)	89 (27.05%)	10 (6.37%)	
Moderate fall risk	183 (37.65%)	149 (45.29%)	34 (21.66%)	
High fall risk	204 (41.98%)	91 (27.66%)	113 (71.97%)	
Nutritional status				<0.001
Normal	194 (39.92%)	171 (51.98%)	23 (14.65%)	
At risk of malnutrition	244 (50.21%)	150 (45.59%)	94 (59.87%)	
Malnourished	48 (9.88%)	8 (2.43%)	40 (25.48%)	
Anxiety				<0.001
Minimal anxiety	234 (48.15%)	192 (58.36%)	42 (26.75%)	
Mild anxiety	179 (36.83%)	110 (33.43%)	69 (43.95%)	
Moderate anxiety	73 (15.02%)	27 (8.21%)	46 (29.30%)	
Depression				0.004
No	66 (13.58%)	55 (16.72%)	11 (7.01%)	
Yes	420 (86.42%)	274 (83.28%)	146 (92.99%)	
ADL dependence				<0.001
No	177 (36.42%)	161 (48.94%)	16 (10.19%)	
Yes	309 (63.58%)	168 (51.06%)	141 (89.81%)	
WBC [10 ~ 9/L, M (P25, P75)]	7.49 [6.04; 9.77]	7.49 [6.06; 9.78]	7.48 [6.04; 9.74]	0.991
RBC [10 ~ 12/L, M (P25, P75)]	4.34 [3.93; 4.78]	4.41 [4.02; 4.82]	4.17 [3.83; 4.62]	<0.001
HGB [g/L, M (P25, P75)]	131.00 [121.00; 141.00]	132.00 [122.00; 142.00]	128.00 [119.00; 141.00]	0.034
PLT [10 ~ 9/L, M (P25, P75)]	206.50 [164.00; 244.00]	208.00 [167.00; 246.00]	205.00 [161.00; 240.00]	0.450
NA [mmol/L, M (P25, P75)]	139.00 [137.00; 142.00]	139.00 [137.00; 142.00]	139.00 [136.00; 142.00]	0.343
Cr [μmol/L, M (P25, P75)]	78.00 [62.90; 93.02]	77.00 [62.80; 90.10]	79.20 [63.40; 102.00]	0.083

BMI, body mass index; CCI, Charlson Comorbidity Index; ADL, activities of daily living; WBC, white blood cell; RBC, red blood cell; HGB, hemoglobin; PLT, platelet; NA, sodium; Cr, creatinine.

### 3.2 Screening variables for frailty in older adults with multimorbidity

**Figure 1:**
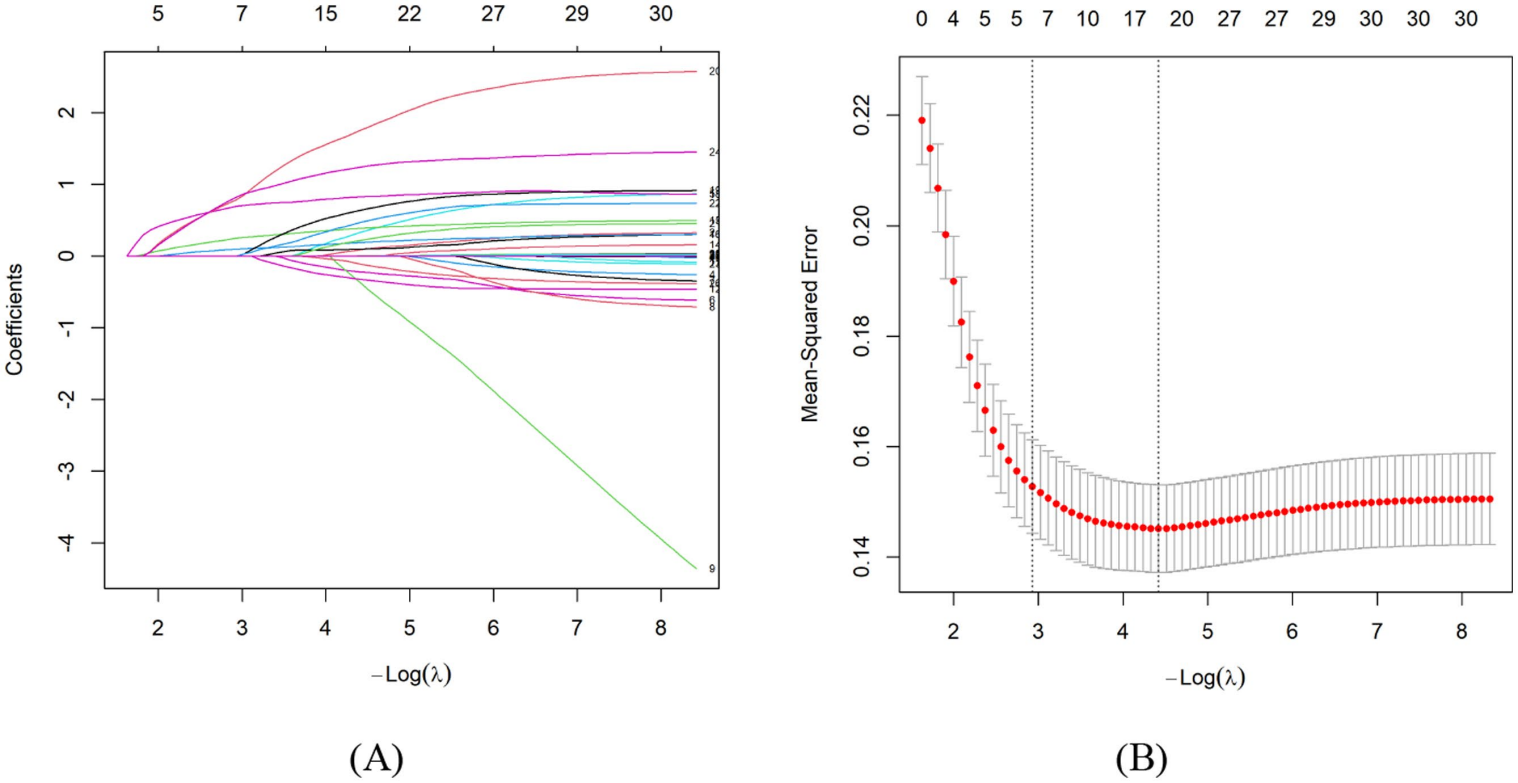
**(A)** LASSO regression screening variables. **(B)** The selection of optimal *λ* by cross—validation.

Using 10-fold cross-validation, the optimal penalty parameter was selected at the 1-SE criterion (λ_{1se} = 0.053). At this value, LASSO identified six factors with non-zero coefficients: income, number of medications, Charlson Comorbidity Index (CCI), fall risk, nutritional status, anxiety, and ADL dependence (Figure 1).

### 3.3 Binary logistic regression analysis of factors associated with frailty in older adults with multimorbidity

Binary logistic regression was conducted using the factors selected by LASSO (Table 2). The number of medications (OR = 1.553, 95% CI: 1.313–1.838) and CCI score (OR = 1.219, 95% CI: 1.001–1.484) were positively associated with frailty. High fall risk (OR = 2.680, 95% CI: 1.014–7.084) was significantly associated with frailty. Nutritional status was strongly associated with frailty, with higher odds among participants at risk of malnutrition (OR = 2.429, 95% CI: 1.344–4.389) and those who were malnourished (OR = 8.235, 95% CI: 3.112–21.788). Mild anxiety (OR = 1.781, 95% CI: 1.016–3.122) and moderate anxiety (OR = 2.100, 95% CI: 1.016–4.339) were associated with increased frailty. ADL dependence was also an independent factor associated with frailty (OR = 4.069, 95% CI: 1.876–8.826). All variables showed low multicollinearity, with VIF values ranging from 1.02 to 1.18 (Table 3).

**Table 2:** Binary logistic regression analysis for frailty in older adults with multimorbidity.

Variable	*β*	Wald	*P*	OR	95%CI
Number of medications	0.440	26.293	<0.001	1.553	1.313 ~ 1.838
CCI	0.198	3.872	0.049	1.219	1.001 ~ 1.484
Fall risk					
Low fall risk				1	
Moderate fall risk	0.178	0.129	0.720	1.195	0.453 ~ 3.153
high fall risk	0.986	3.950	0.047	2.680	1.014 ~ 7.084
Nutritional_status					
Normal				1	
At risk of malnutrition	0.887	8.638	0.003	2.429	1.344 ~ 4.389
Malnourished	2.108	18.039	<0.001	8.235	3.112 ~ 21.788
Anxiety					
Minimal anxiety				1	
Mild anxiety	0.577	4.066	0.044	1.781	1.016 ~ 3.122
Moderate anxiety	0.742	4.012	0.045	2.100	1.016 ~ 4.339
ADL dependence	1.403	12.617	<0.001	4.069	1.876 ~ 8.826

CCI, Charlson Comorbidity Index; ADL, activities of daily living.

**Table 3:** Variance inflation factors for variables included in the multivariable model.

Variable	VIF
Number of medications	1.088525
CCI	1.056618
Fall risk	1.089621
Nutritional status	1.024138
Anxiety	1.033309
ADL dependence	1.184653

BMI, body mass index; CCI, Charlson Comorbidity Index; ADL, activities of daily living.

### 3.4 Construction of a nomogram for frailty in older adults with multimorbidity

**Figure 2:**
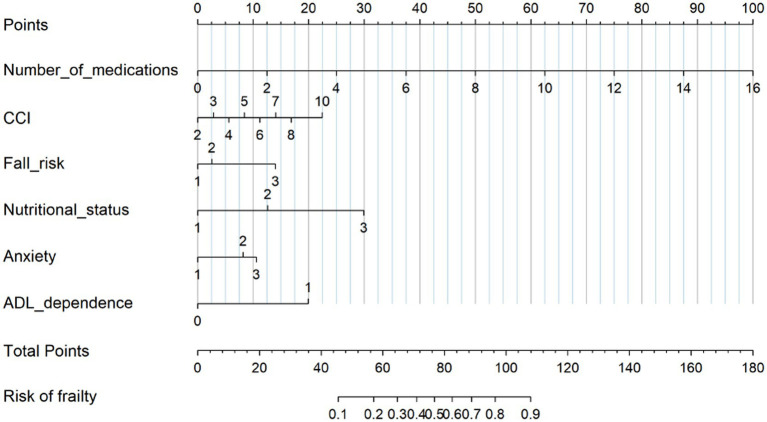
Nomogram for predicting the frailty status in older ED patients with multimorbidity. Odds ratios (ORs) were estimated using multivariable logistic regression. Reference categories were low fall risk, normal nutritional status, minimal anxiety, and no ADL dependence. Fall risk as low fall risk = 1, moderate fall risk = 2, high fall risk = 3; nutritional status as normal = 1, at risk of malnutrition = 2, malnourished = 3; anxiety as minimal = 1, mild = 2, moderate = 3; ADL dependence as no = 0, yes = 1. ORs and 95% confidence intervals (CIs) are reported; *p*-values are two-sided.

A nomogram was developed based on the final multivariable logistic regression model to estimate the individual probability of frailty (Figure 2). The model incorporated six predictors: number of medications, CCI, fall risk, nutritional status, anxiety, and ADL dependence. Each variable was assigned a point value, and the total score was used to estimate the probability of the current frailty status. Higher total scores indicated a greater likelihood of frailty.

### 3.5 Validation and assessment of the nomogram for frailty in older adults with multimorbidity

#### 3.5.1 Discrimination performance of the prediction model

**Figure 3:**
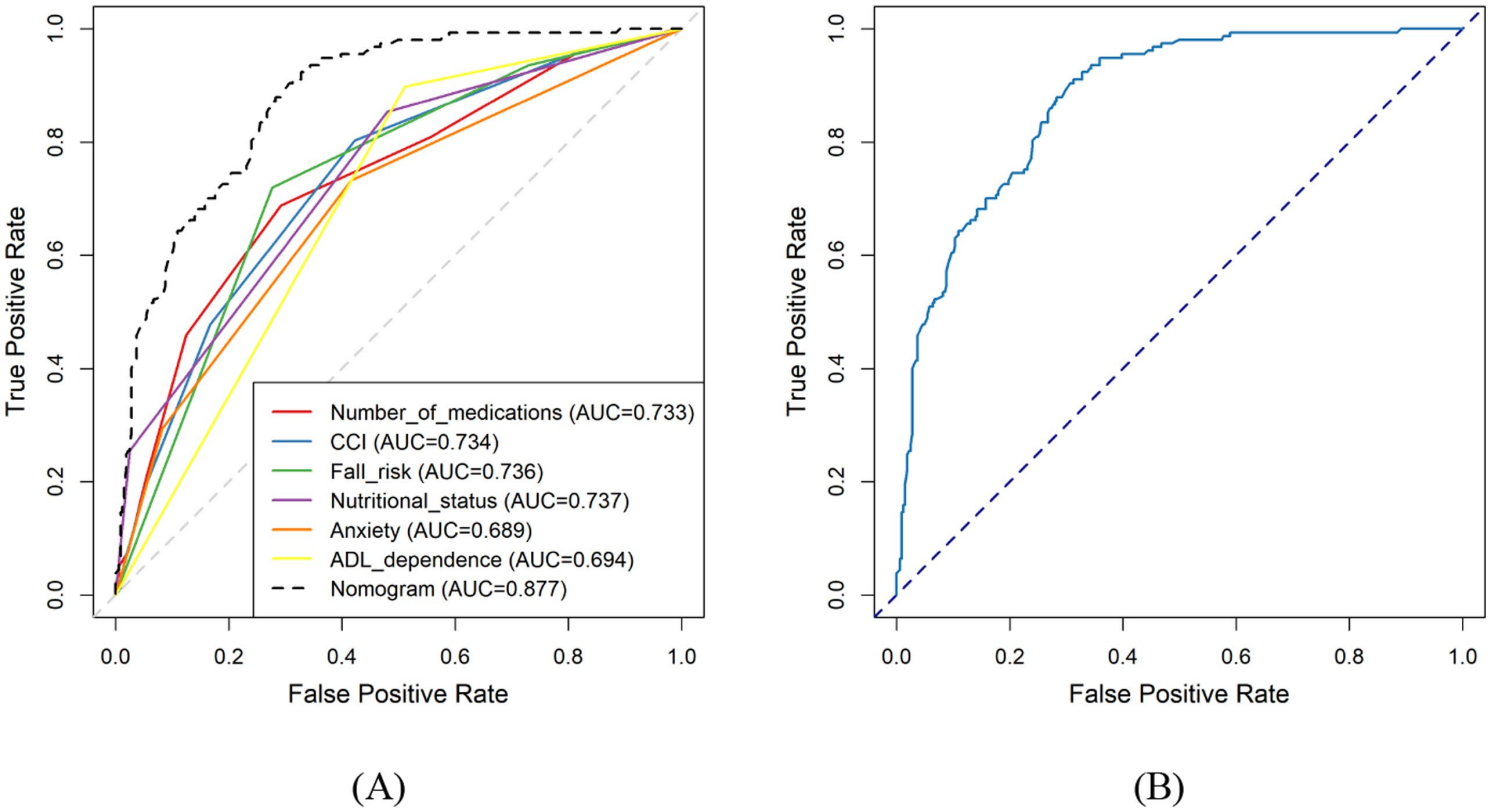
**(A)** ROC curves of individual predictors and the nomogram model. **(B)** ROC curves of the nomogram model.

The discrimination ability of each predictor and the final nomogram was assessed using ROC curves (Figure 3A). Among the individual variables, CCI (AUC = 0.734), nutritional status (AUC = 0.737), number of medications (AUC = 0.733), and fall risk (AUC = 0.736) demonstrated moderate discrimination. Anxiety (AUC = 0.689) and ADL dependence (AUC = 0.694) showed lower discriminatory ability. The nomogram achieved the highest discrimination, with an AUC of 0.877, indicating good discrimination and generalizability (Figure 3B).

#### 3.5.2 Calibration

**Figure 4:**
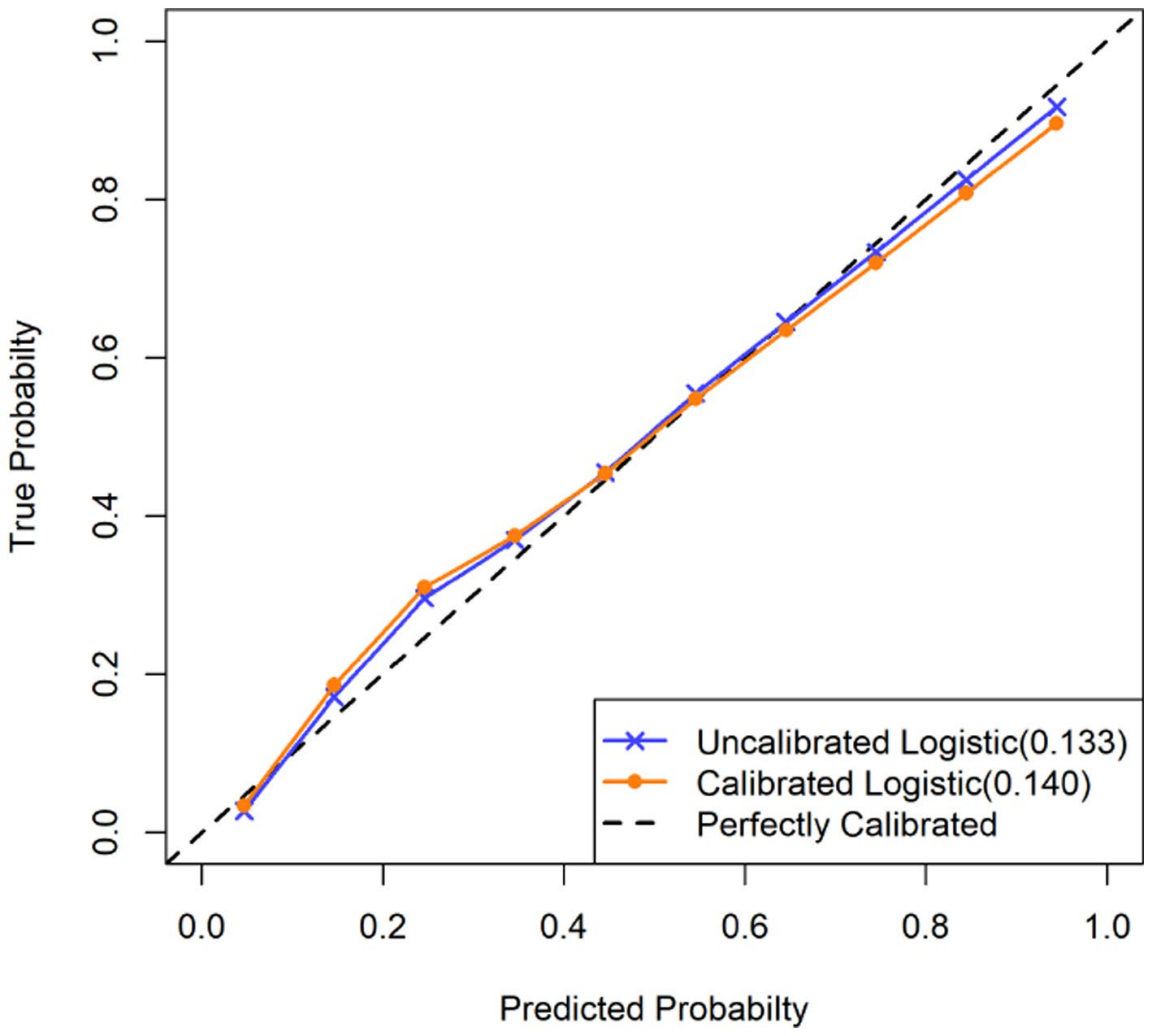
Calibration curve of the nomogram.

The Hosmer-Lemeshow test showed no significant lack of fit in either the training set (*χ*^2^ = 8.876, *p* = 0.262). After 1,000 bootstrap resamples, the calibration curve closely aligned with the diagonal reference line, indicating good agreement between the predicted and observed probabilities. The calibration intercept and slope were −0.028 and 0.926. The Brier scores were 0.133 and 0.140 for the uncalibrated and calibrated logistic models, respectively. All Brier scores were below 0.25, indicating acceptable calibration performance. The calibration curves (Figure 4) also showed good agreement between the predicted and observed probabilities in both datasets.

#### 3.5.3 Clinical utility

**Figure 5:**
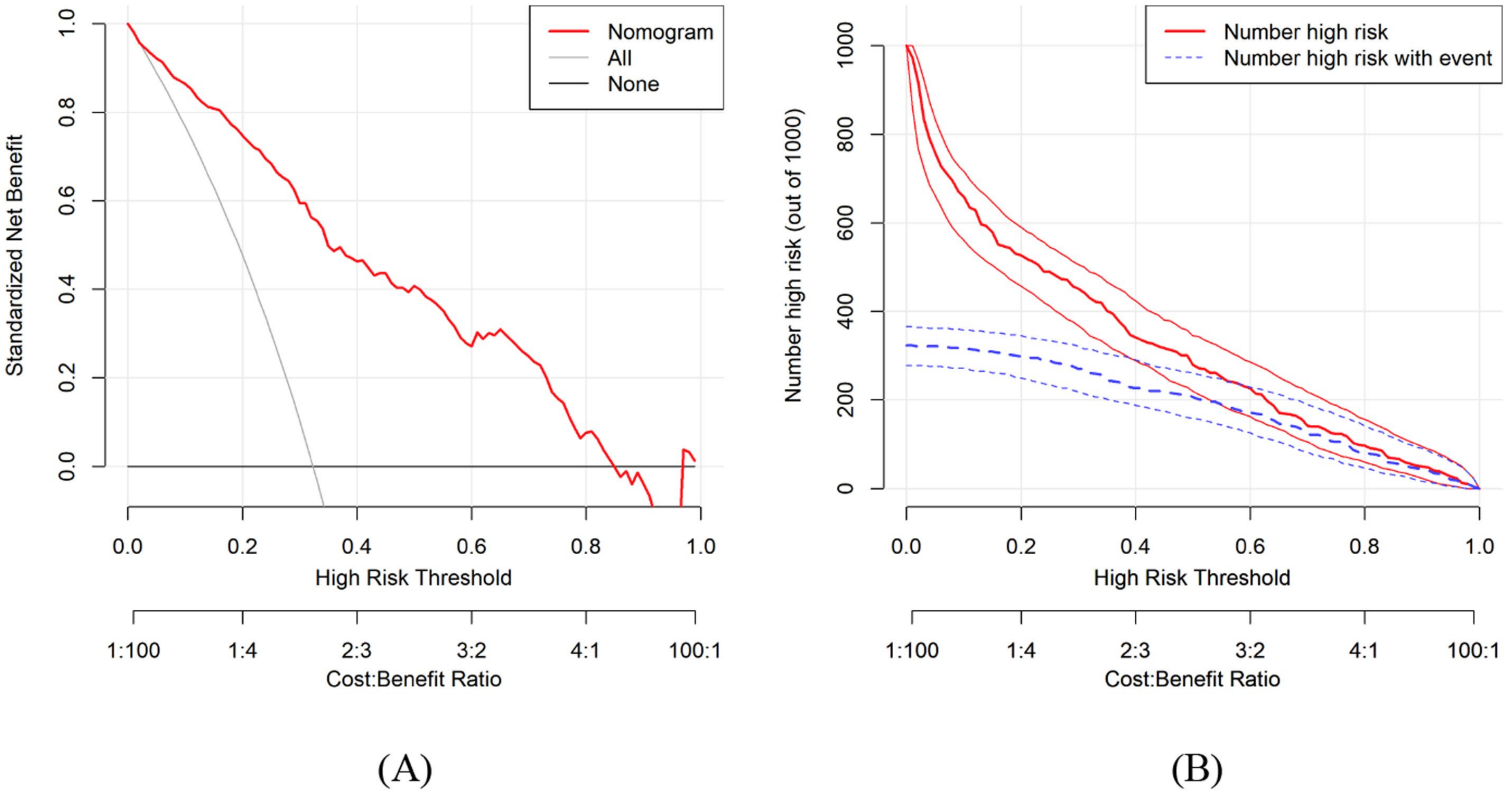
**(A)** Decision curve analysis (DCA) of the nomogram. **(B)** Clinical impact curve (CIC) of the nomogram.

After 1,000 bootstrap resamples, the DCA curve showed that within threshold probabilities of 1, 3–84%, and 97–99%, the model provided a higher net benefit across most threshold probabilities than the “treat-all” and “treat-none” strategies, indicating good clinical utility of the model (Figure 5A). The CIC curves showed that the model effectively identified high-risk patients, notably when the threshold probability exceeded 0.80 in both the training and validation sets (Figure 5B).

## 4 Discussion

Our study shows that the prevalence of frailty in older patients with multimorbidity in the emergency department was 32.3%. Using Least Absolute Shrinkage and Selection Operator (LASSO) regression and multivariate logistic regression, we identified fall risk, nutritional status, number of medications, Charlson Comorbidity Index (CCI), anxiety, and dependence in Activities of Daily Living (ADL) as independent factors associated with frailty.

In our study, fall risk was significantly associated with frailty. Falls were recognized as a common adverse outcome among older adults with frailty and pre-frailty (33). Frail older adults are more vulnerable to falls due to reduced muscle strength, impaired balance, slower gait speed, and diminished physiological reserve (34, 35). Conversely, falls may lead to fractures, hospitalization, disability, loss of independence, and even mortality, which may in turn accelerate the progression of frailty (36). Furthermore, fear of falling is common among older adults after a fall event or when they perceive themselves to be at high risk of falling (37). This fear may result in activity restriction, reduced mobility, and avoidance of daily movement, ultimately contributing to physical deconditioning, muscle loss, and worsening frailty (38). This association may be explained by a bidirectional relationship between falls and frailty. Therefore, effective fall risk assessment and timely intervention may be essential components of frailty prevention and management in older adults.

Medication count was independently associated with frailty (OR = 1.553), indicating a 55.3% increase in odds with each additional medication. This is consistent with prior evidence that greater medication exposure and polypharmacy (e.g., ≥ six drugs) are associated with frailty in adults aged ≥65 years (39, 40). Although the mechanisms remain incompletely understood (41), higher medication burden may increase adverse drug events, drug–drug interactions, and overall treatment burden, thereby accelerating functional decline. Significantly, clinical medication reviews in older adults with polypharmacy may reduce ED visits (42), supporting medication optimization as a pragmatic component of frailty assessment and management.

Comorbidity burden, captured by the Charlson Comorbidity Index (CCI), also remained independently associated with frailty, with each 1-point increase corresponding to 21.9% higher odds of FRAIL-defined frailty (OR = 1.219). CCI was initially developed to quantify the comorbidity burden and predict mortality (21, 43) and has also been incorporated into ED triage to support clinical assessment and decision-making. For instance, a multicenter prospective study embedded in the Manchester Triage System developed a nomogram to predict 90-day mortality and identified CCI as an independent predictor alongside functional and vulnerability proxies (e.g., impaired mobility, cognitive impairment, loss of autonomy, ambulance arrival, and recent hospitalization), achieving excellent discrimination after internal validation (AUROC 0.910) (44). Our findings extend this work by directly modeling frailty using the FRAIL scale, aligning with the clinical need to support frailty assessment and individualized management in the ED. Nevertheless, the relationship between comorbidity and frailty is likely complex, and further research is warranted to clarify how specific disease patterns, and their treatments contribute to frailty among multimorbid older adults.

Nutritional status emerged as one of the strongest factors of frailty, with both “at risk of malnutrition” and “malnourished” categories showing substantially elevated odds. Recent evidence indicates that malnutrition is not merely a consequence of frailty but may also drive its onset and progression (45). Unhealthy dietary patterns (46), diets with a higher dietary inflammatory index (47), and higher consumption of ultra-processed foods (48) have been associated with frailty risk factors. This process may accelerate the decline in physiological reserve in older adults through multiple pathways, including protein–energy deficiency (49) and micronutrient inadequacy (50), and disruption of the gut microbiota (51), which may accelerate loss of physiological reserve and promote frailty development. Given its modifiability, brief nutritional screening at ED triage, followed by early dietitian referral and/or initiation of high-protein oral supplements, may be a feasible strategy to mitigate frailty status. Longitudinal studies are needed to determine whether ED-initiated nutritional interventions can reverse or stabilize frailty trajectories in high-risk populations.

Anxiety was another independent correlate of frailty, underscoring the bidirectional relationship between psychological distress and physical resilience. Prior evidence similarly suggests a higher burden of anxiety among frail older adults (52) and increased odds of frailty among individuals with anxiety symptoms (53). However, effect sizes may vary across populations and measurement approaches. Anxiety can present with insomnia, reduced appetite, and diminished motivation for physical activity (54), all of which may contribute to declines in muscle strength and endurance. Chronic anxiety has also been linked to hypothalamic–pituitary–adrenal axis dysregulation and heightened sympathetic activity, potentially accelerating cardiovascular and metabolic damage (55). Conversely, frailty may provoke anxiety through fear of falling, dependence, and concerns about future disability (56). In the ED, brief screening for anxiety may help identify patients who could benefit from timely mental-health assessment and follow-up, and future work should evaluate whether integrating psychological support into frailty pathways improves both emotional well-being and functional outcomes.

The ADL dependence was an independent factor of frailty in our study, emphasizing functional limitation as a key sign of decreased physiological reserve. Evidence from the China Health and Retirement Longitudinal Study (CHARLS) shows that ADL dependency forecasts incident frailty over 2 years, with a strong link between ADL limitation and frailty (OR = 5.658, 95% CI: 4.278–7.483) (57). Similarly, ADL has also been reported to be associated with frailty among older patients with ischemic stroke (OR = 7.494, 95% CI: 4.156–13.514) (13). These results support the need to include quick functional assessments in early frailty screening, especially in the ED, where prompt decisions about monitoring, geriatric evaluation, rehabilitation, and discharge planning are crucial.

We developed a logistic regression model to identify frailty among older ED patients with multimorbidity using six routinely assessed variables (fall risk, nutritional status, medication count, CCI, anxiety, and ADL dependence). The model showed good discrimination (AUC = 0.877). This performance compares favorably with previously reported models in inpatient multimorbidity cohorts (AUC = 0.736) (9) and community samples (AUC = 0.777 and 0.756 in training and validation, respectively) (58) while being tailored to the ED context, where timely frailty assessment is particularly important. To facilitate bedside implementation, we translated the model into a nomogram for identifying current frailty status at ED presentation. Because all six variables are available from routine triage and initial bedside assessment, the nomogram can be applied immediately without additional testing. Its intuitive visual format supports the prompt recognition of patients with frailty, and helps inform closer monitoring, comprehensive geriatric assessment, and timely multidisciplinary management, with minimal disruption to ED workflows.

### 4.1 Strengths and limitations

This study has several methodological strengths. First, LASSO regression enabled robust variable selection and reduced the risk of overfitting. Second, the model incorporated multidimensional variables—clinical, functional, nutritional, and psychological—reflecting the multifactorial nature of frailty. Third, the model was validated on a random hold-out set, demonstrating its robustness and reproducibility. Finally, the DCA and CIC analyses confirmed the model’s clinical applicability, suggesting that it may support frailty screening and assessment among older adults in emergency or acute care settings.

Several limitations should be acknowledged. First, this study was conducted at a Chinese emergency department, which may limit the generalizability of the findings to other populations, healthcare systems, and cultures. Second, the cross-sectional design precludes causal inference about the relationship between the factor and frailty. Some variables, particularly psychological and functional assessments, were based on self-report or subjective judgment, which may introduce measurement bias. Third, although eligible patients were recruited consecutively during the study period, patients with severe cognitive impairment and critical illness were excluded, which may have introduced selection bias and reduced the representativeness of the sample by underrepresenting more vulnerable or clinically unstable older adults. Therefore, the model may be more applicable to relatively stable older adults with multimorbidity presenting to the ED. In addition, because frailty was assessed at ED presentation, some FRAIL scale components, such as fatigue, resistance, and ambulation, may have been influenced by the acute illness state rather than baseline functional status alone. As a result, the measured frailty status may partly reflect transient illness-related impairment in addition to underlying frailty. Finally, although the model performed well in internal validation, external validation across multiple centers and diverse populations is needed.

Future studies should explore external validation of the proposed predictive model in multi-center, prospective cohorts. Such validation efforts should encompass diverse healthcare systems and patient populations to rigorously assess the model’s generalizability, calibration, and clinical transportability. Furthermore, integrating objective biomarkers (e.g., inflammatory markers), performance-based measures (e.g., gait speed), and longitudinal follow-up data could enhance the model’s predictive accuracy and provide deeper insights into the causal pathways underlying frailty in this vulnerable population.

## 5 Conclusion

This study developed and validated a nomogram to identify frailty among older adults with multimorbidity in the emergency department. The model demonstrated good discrimination, calibration, and clinical utility, providing a rapid, practical tool for frailty screening and assessment in time-sensitive ED settings. Further external validation across diverse emergency care environments is warranted.

## Data Availability

The original contributions presented in the study are included in the article/supplementary material, further inquiries can be directed to the corresponding author.
